# Supplementary material for: Nonequilibrium polysome dynamics promote chromosome segregation and its coupling to cell growth in Escherichia coli
Source: bioRxiv. 2025 Mar 15:2024.10.08.617237. Preprint. [Version 3] doi: 10.1101/2024.10.08.617237 (PMC11952301; doi:10.1101/2024.10.08.617237)
Supplement: Supplement 7 [file NIHPP2024.10.08.617237v3-supplement-7.pdf]

bioRxiv preprint doi: https://doi.org/10.1101/2024.10.08.617237; this version posted March 15, 2025. The copyright holder for this preprint (which was not certified by peer review) is the author/funder, who has granted bioRxiv a license to display the preprint in perpetuity. It is made available under aCC-BY 4.0 International license.

SUPPLEMENTARY FIGURE LEGENDS

Figure 1 – figure supplement 1: Reproducibility analysis of the dynamic ribosome and nucleoid distributions between microfluidic experiments. While phase-contrast images were acquired every minute in all microfluidics experiments, two different intervals (1 min and 3 min) were used for fluorescence image acquisition. **A.** Kymographs showing the RplA-GFP and HupA-mCherry concentration in CJW7323 cells growing in microfluidic channels in M9gluCAAT. Cell and nucleoid contours are shown in each channel using a different color for each cell lineage (from dark purple to bright orange). **B-E.** Two biological replicate experiments were performed for this strain and nutrient condition using different intervals of fluorescence image acquisition (1 or 3 min). **B.** Plots showing that the instantaneous and average division cycle growth rates were nearly identical between the two experiments. **C.** Plots showing the distributions of the indicated fluorescence signals for the 1-min acquisition interval (black) compared to the 3-min frame rate interval (orange) before and after correction. The difference in the excitation power between the two experiments was 50% (240 %ms vs. 360 %ms), which was also reflected in the ratio of their average fluorescence values

bioRxiv preprint doi: https://doi.org/10.1101/2024.10.08.617237; this version posted March 15, 2025. The copyright holder for this preprint (which was not certified by peer review) is the author/funder, who has granted bioRxiv a license to display the preprint in perpetuity. It is made available under aCC-BY 4.0 International license.

(1.4 for RplA-GFP and 1.6 for HupA-mCherry). The fluorescence values from the 1-min interval experiment were corrected by multiplying by these ratios, resulting in a near-perfect overlap between fluorescence distributions after correction. **D.** Plots showing that the scaled (z-score) RplA-GFP and HupA-mCherry intensity profiles from birth to division were almost identical between the two experiments. The intensity profiles are shown for 10 cell division cycle intervals (1489 and 2633 cell division cycles for the 1-min and 3-min interval experiments, respectively). The z-score was calculated for each segmented cell by subtracting the whole cell average fluorescence and then dividing the difference by the standard deviation. Each intensity profile corresponds to the average z-score of all segmented cell instances within the corresponding cell cycle interval. **E.** Plot showing that the average RplA-GFP concentration from birth to division (averages ± SD across 20 cell division cycle bins) remained constant and was virtually identical between the two experiments.

Figure 1 – figure supplement 2: Determination of the relative timing of cell constriction. The average cell width at mid-cell was used to estimate the relative timing of cell constriction from birth (0%) to division (100%). Considering the variability in the constriction onset across single cells, the average timing corresponds to the elbow of the curve, or the point with the maximum distance from the linear segment that connects the ends of the cell width curve, also known as the chord.

Figure 1 – figure supplement 3: Intracellular distributions of RplA-GFP in rifampicin-treated cells following rifampicin removal. A. Representative image of RplA-GFP fluorescence in cells (CJW7323) treated with rifampicin (100 µg/mL). The cells were treated for 45 min, washed, and spotted on an M9gluCAAT agarose pad without antibiotic. The presented snapshot corresponds to the first timepoint immediately after cell spotting showing diffuse distribution of RplA-GFP signal. **B.** Time-lapse fluorescence (fluor.) images showing the emergence of RplA-GFP signal accumulation at mid-cell (single arrow) or quarter cell positions (double arrows) during recovery from rifampicin treatment (same experiment as in panel A).

Figure 1 – figure supplement 4: Tracking nucleoid segregation cycles. Since the timing of nucleoid segregation varied between cell division cycles, the nucleoid segregation cycles were tracked independently of the cell division cycles for each cell lineage to measure the relative timing of RplA-GFP accumulation and HupA-mCherry depletion in the middle of the nucleoid (as shown in Figure 1E). **A.** Plots showing the frequency of cells with one, two, three, or four detected nucleoid objects across 10 cell division cycle bins from birth (top) to division (bottom). Data from 4122 cell division cycles are shown. **B.** The polarity of the cells (+/-) and the relative position of the nucleoid mask (toward the new or old pole) was used to track the nucleoid segregation cycle. This strategy was used to identify four groups of nucleoid segregation cycles (group -2, -1, 1 and 2). Group -2 includes nucleoids that were “born” toward the old cell pole in a mother cell with negative (-) polarity and were inherited at the center of a daughter cell with a negative

bioRxiv preprint doi: https://doi.org/10.1101/2024.10.08.617237; this version posted March 15, 2025. The copyright holder for this preprint (which was not certified by peer review) is the author/funder, who has granted bioRxiv a license to display the preprint in perpetuity. It is made available under aCC-BY 4.0 International license.

polarity. Group -1 includes nucleoids that were “born” toward the new cell pole in a mother cell with negative (-) polarity and were inherited at the center of a daughter cell with a positive (+) polarity. Group 1 includes nucleoids that were “born” toward the new cell pole in a mother cell with positive (+) polarity and were inherited at the center of a daughter cell with negative (-) polarity. Group 2 includes nucleoids that were “born” toward the old cell pole in a mother cell with positive (+) polarity and were inherited at the center of a daughter cell with positive (+) polarity. **C.** Plots showing the distributions of nucleoid positions around the cell center for 10 nucleoid segregation cycle bins, from the time a nucleoid was “born” (top) until it split (bottom). These plots show the inheritance of the nucleoids from the quarter cell positions of the mother cells to the middle of their daughters for the four groups of nucleoid segregation cycles. The solid gray line represents the average density of the nucleoid positions for all four groups. Data from 2286 complete nucleoid cycles are shown. **D.** Schematic that explains the definition of a nucleoid cycle. The nucleoid cycle ranges from the end of a nucleoid splitting event, until the next splitting of the sister nucleoids. It usually extends beyond cell division, into the next cell division cycle.

Figure 1 – figure supplement 5: Correlations used to calculate the relative contribution of polysome accumulation and cell elongation to nucleoid migration. A. Correlation (Spearman ρ = 0.47, p-value < 10^{-10}) between the rate of RplA-GFP accumulation at mid-cell ($\frac{\Delta RplA_{mid-cell\ conc.}}{\Delta T}$) and the rate of distance increase between the sister nucleoids minus the rate of cell elongation ($\frac{\Delta Distance_{nuc}}{\Delta T} - \frac{\Delta Length_{cell}}{\Delta T}$) across cells. The colormap corresponds to a Gaussian kernel density estimation. Binned data are also shown (mean ± SEM, 75 to 177 cell division cycles per bin, 9 bins in total) within the 5th-95th percentiles of the *x*-axis range. **B.** Scaled correlations (z-scores) between $\frac{\Delta RplA_{mid-cell\ conc.}}{\Delta T}$ (blue) or $\frac{\Delta Length_{cell}}{\Delta T}$ (grey) and $\frac{\Delta Distance_{nuc}}{\Delta T}$ (y-axis) during four relative time bins (1335 to 1957 cell division cycles per bin) covering the period from the end of nucleoid splitting until cell division. Shown are the Spearman correlations (ρ), all with a p-value below 10^{-6}, except for the one marked with an asterisk (p-value = 0.02).

Figure 1 – figure supplement 6: Examination of the relative timing of the initiation of nucleoid constriction and the accumulation of polysomes at mid-nucleoid. A. RplA-GFP and HupA-mCherry concentration (fluorescence arbitrary units) images of four representative single cells (CJW7323) growing in M9glyT and their fluorescence intensity profiles along the cell length. **B.** Demographs of the scaled RplA-GFP and HupA-mCherry concentration from 13554 *E. coli* cells grown in M9glyT. **C.** Average 1D intensity profiles of the scaled (divided by the whole cell average) RplA-GFP and HupA-mCherry concentration for 12 bins of cells lengths (>1100 cells per intensity profile). **D.** Plot showing the scaled RplA-GFP and HupA-mCherry concentration in the middle of the cells for increasing cell length (~370 cells per bin, 25 bins). Cells longer than 3 μm were excluded to avoid the effects of cell constriction. The arrow and vertical dashed line indicate the minimum cell length bin with an apparent HupA-mCherry depletion at mid-cell, marking the initiation of nucleoid splitting. The error bars indicate mean ± standard error of the mean (SEM).

bioRxiv preprint doi: https://doi.org/10.1101/2024.10.08.617237; this version posted March 15, 2025. The copyright holder for this preprint (which was not certified by peer review) is the author/funder, who has granted bioRxiv a license to display the preprint in perpetuity. It is made available under aCC-BY 4.0 International license.

Figure 2 – figure supplement 1: Demographs of scaled ribosome and nucleoid fluorescence for strains with different ribosome markers and under various nutrient conditions. Demographs are ordered according to increasing average cell area, which scales with growth rate (Schaechter et al., 1958), from left to right and top to bottom. These demographs were constructed from snapshots of the following strains: **A.** CJW6768 (RplA-mEos2 ribosomal marker, 747 to 2446 cells per condition), **B.** CJW6769 (RpsB-mEos2 ribosomal marker, 690 to 3169 cells per condition), **C.** CJW7020 (RplA-msfGFP ribosomal marker, 657 to 2432 cells per condition) and **D.** CJW7021 (RpsB-msfGFP ribosomal marker, 788 to 1950 cells per condition), using the cell length as a proxy for the cell division cycle, with the signal intensity profile sorted from the shortest newborn cells to the longest predivisional cells. Note that the demographs for the CJW6768 strain (panel A) in the nutrient conditions M9mann, M9mala, M9malt, M9mannCAAT, M9glyCAAT and M9malaCAAT are not shown here, as they are presented in Figure 2A. The nutrient abbreviations and compositions are explained in Table S1.

Figure 2 – figure supplement 2: Correlation of the extent and relative timing of polysome accumulation with nucleoid segregation at the single-cell level. A. Plot showing the variability in growth rate (GR) across cell division cycles for cells growing in microfluidics in M9gluCAAT (4114 cell division cycles, 9 growth rate bins with 93 to 860 cell division cycles each). **B.** Ensemble kymographs of the RplA-GFP and HupA-mCherry concentration normalized by the average fluorescence for the slowest, intermediate and fastest growing population bins shown in panel A. **C.** Plot showing the correlation between the average RplA-GFP accumulation and HupA-mCherry depletion at mid-cell across the growth rate bins (mean ± SD, 93 to 860 cell division cycles per bin) shown in panel A.

Figure 2 – figure supplement 3: Calculation of RplA-msfGFP concentration after cell curvature correction. A. Average 2D RplA-msfGFP projections for short (2.5 to 3 µm – left), unconstricted cells and long (3.5 and 5.5 µm - right) constricted cells. The average RplA-msfGFP signal from 1000 sampled cells (CJW7651) is shown for each population. **B.** The 2D cell areas were divided in cylindrical sectors with a height (h) of a single pixel. The number of cylindrical sectors corresponds to the average cell length in pixels (40 and 60 sectors for the unconstricted and constricted cells, respectively). The radius (r) of each cylindrical segment corresponds to half the cell diameter for that specific cell length position. The cylindrical segments at the poles or constriction site have a smaller radius due to the curvature of the cell boundaries. The division of the cell area into cylindrical segments allowed us to calculate the volume for each segment ($V_{cylinder} = \pi\, r^2\, h$). The sum of the fluorescence per segment was corrected for the 3D curvature of the cell boundaries by dividing by the volume of the cylinder, either considering the entire cell width or different depths of view narrower than the maximal cell width. **C.** One-dimensional profiles of the cylinder diameter are also indicative of the cell curvature at the poles and constriction site. **D.** Comparisons of the 1D average RplA-msfGFP projection between the poles before (dashed lines) or after correcting for the cell curvature

bioRxiv preprint doi: https://doi.org/10.1101/2024.10.08.617237; this version posted March 15, 2025. The copyright holder for this preprint (which was not certified by peer review) is the author/funder, who has granted bioRxiv a license to display the preprint in perpetuity. It is made available under aCC-BY 4.0 International license.

(solid lines) for different depths of view (colormap). The uncorrected signal (dashed lines) corresponds to the average fluorescence along each cylindrical segment (mean of pixel intensities). The 1D profiles were normalized by dividing by the maximal RplA-msfGFP concentration at mid-cell. This correction shows that the pronounced decrease of the RplA-msfGFP fluorescence at the poles is the result of the curvature of the cell boundaries. The observed over-correction at the cell poles for larger depths of view (> 8 pixels or px) is likely due to the over-estimation of the cell segmentation mask that was determined based on the phase contrast snapshot images of cells on agarose pads.

Figure 3 – figure supplement 1: Quantification of the polysome and nucleoid asymmetries using fitted Gaussian functions. A. Example images of RplA-GFP and HupA-mCherry fluorescence signals in a single newborn cell instance. **B.** Plots showing the Gaussian-function fitting on RplA-GFP and HupA-mCherry intensity profiles from the single cell snapshot shown in panel A. Three Gaussian functions were fitted to the RplA-GFP fluorescence in newborn cells (eq. 11 in Methods), capturing the accumulation of polysomes at mid-cell and the poles. Two Gaussian functions were fitted to the HupA-mCherry fluorescence (eq. 12 in methods) capturing the two lobes of the segregating sister nucleoids. The parameters of the fitted Gaussian functions provided information about the position (μ: mean) and the concentration (A: amplitude) of each fluorescence statistic, as well as the cell length range occupied by it (σ: standard deviation). The Gaussian area (eq. 13 in Methods) corresponds to the abundance of each macromolecule. The RplA-GFP Gaussians were fitted above the cellular background, which presumably corresponds to the uniform fluorescence of free ribosomes or ribosomal subunits. For the HupA-mCherry Gaussian fittings, the cellular background corresponds to the DNA-free cell regions. The Gaussian parameters were also used to describe the polysome asymmetries between the poles (eq. 14 in Methods) as well as the position of the nucleoid around the cell center (eq. 15 in Methods). These statistics were used in Figure 3B. **C.** Gaussian functions were also fitted to the ensemble average RplA-GFP and HupA-mCherry fluorescence (right) calculated from all the cell segmentation instances of a single cell division cycle, 0-10% from birth to division (left). **D.** The Gaussian fitting to the ensemble RplA-GFP and HupA-mCherry intensity profiles (concentration in arbitrary fluorescence units) early in the cell division cycle for the 2D projection shown in panel C (right).

Figure 3 – figure supplement 2: Correlations between the polysome distribution statistics and the nucleoid compaction asymmetries. A. Plots showing the nucleoid and polysome density axial asymmetry determined using the parameters of the fitted Gaussians on the HupA-mCherry and RplA-GFP fluorescence (eq. 16 and eq. 18 in Methods). **B.** Plots showing the scaled correlations between the polysome Gaussian parameters (μ, A, and σ for each polysome accumulation) and the nucleoid density asymmetry (eq. 16 in Method; 2103 cell division cycles). Each marker corresponds to the data for a single cell division cycle (0-10% into the cell division cycle) as in Figure 3 – figure supplement 1C-D. A Gaussian kernel density estimation (KDE) was used to illustrate the density of the scatter plots (see Methods). **C.** Bar graph showing

bioRxiv preprint doi: https://doi.org/10.1101/2024.10.08.617237; this version posted March 15, 2025. The copyright holder for this preprint (which was not certified by peer review) is the author/funder, who has granted bioRxiv a license to display the preprint in perpetuity. It is made available under aCC-BY 4.0 International license.

the coefficients of a linear mixed-effects model (see eq. 17 in Methods) used to identify the polysome statistics that contribute to the nucleoid density asymmetry. The highly contributing polysome statistics were combined into a compound polysome statistic (eq. 18 in Methods) that describes the available DNA space between polysome accumulations. This compound statistic correlates with the nucleoid density asymmetry (eq. 16 in Methods) as shown in Figure 3F. The stars indicate the polysome statistics that most significantly correlate with the nucleoid density asymmetry (absolute coefficient above 0.3).

Figure 4 – figure supplement 1: Effects of chloramphenicol treatment on nucleoids in non-dividing cells. A. Representative example of an elongated cephalexin-treated cell that does not fuse its nucleoids upon chloramphenicol treatment. **B.** Representative example of an elongated cephalexin-treated cell that fuses two of its nucleoids upon chloramphenicol treatment. **C.** Plot showing the distributions of the minimum distance between adjacent nucleoids in cells where nucleoid fusion happens (n = 67), compared to cells that do not display nucleoid fusion (n = 134). The two subpopulations were classified as described in the Methods section.

Figure 4 – figure supplement 2: Determination of simulated cell growth parameters based on experimental measurements. Polysome and nucleoid dynamics were simulated for 10 different growth rates. The predivisional polysome and nucleoid profiles of simulated trajectories initialized from the steady state (Figure 4A) were used as initial conditions (D_n = 10^{-3} µm^2/s). The cell length at birth increased with growth rate according to published measurements (Govers et al., 2024).

Figure 4 – figure supplement 3: Comparison of cell length at nucleoid splitting between simulations and experimental data. Across different growth rates, the cell lengths at nucleoids splitting are compared between our model simulations and the experimentally determined population-averaged statistics (Govers et al., 2024). The range of the y-axis spans 2 µm (between 3 and 5 µm) to match the ~2 µm range of the cell areas at birth across nutrient conditions, which spans between 2 and 4 µm in Figure 4 – figure supplement 2.

Figure 4 – figure supplement 4: Simulation of an extended model with three different polysome species. Kymographs of simulated data of an extended version of the theoretical model that considers three polysome species with different diffusion coefficients $D_p = 0.018,\ 0.023, or\ 0.028\ \mu\mathrm{m}^2/s$, representing polysomes with a decreasing number of ribosomes.

Figure 6 – figure supplement 1: Phenotypic effects of transcription inhibition. Plots showing cellular parameters (instantaneous growth rate, cell area, nucleoid area, and the ratio between nucleoid area and cell area, or NC ratio) affected by the rifampicin treatment. The instantaneous growth rate was estimated

bioRxiv preprint doi: https://doi.org/10.1101/2024.10.08.617237; this version posted March 15, 2025. The copyright holder for this preprint (which was not certified by peer review) is the author/funder, who has granted bioRxiv a license to display the preprint in perpetuity. It is made available under aCC-BY 4.0 International license.

using the log-transformed cell area, using a rolling window of 10 min. The black line corresponds to the averages from 1859 cell division cycles and the gray-shaded area indicates the range of one standard deviation around the mean. The two cycles of rifampicin treatment (as shown in Figure 5A) were overlaid for this plot, using the time of rifampicin addition as t = 0 min.

Figure 7 – figure supplement 1: DNA content during protein overexpression from plasmids. Plots showing the scaling between the DNA content (DRAQ5 fluorescence area) and the cell size (side scatter area) quantified by flow cytometry, comparing uninduced and induced (100μM IPTG for 200 min) conditions for CJW7798 cells. Data from three biological data are shown.

Figure 7 – figure supplement 2: Simulations of ectopic polysome formations away from the nucleoid. Polysome formation was introduced at fixed nucleoid-free regions in the theoretical model to mimic the ectopic polysome formation due to plasmid-driven expression in experiments (as in Figure 7). The model recapitulates the two main phenotypes observed in experiments. The simulations were initialized from the steady-state polysome and nucleoid distribution, similar to the steady state in Figure 4A. **A.** Kymograph of simulated data for which polysomes are ectopically produced near the cell pole, at a rate equivalent to 46% of total polysome production at cell birth. **B.** Kymograph of simulated data for which polysomes are produced between the sister nucleoids, at a rate equivalent to 47% of total polysome production at cell birth.

Figure 7 – figure supplement 3: Phenotypic effects of prolonged protein overexpression from plasmids. Representative phase contrast and fluorescence images of cells (CJW7798) after prolonged induction (100 μM IPTG, for 9 h and 15 min) of mTagBFP2 expression from aT7 promoter on a multi-copy plasmid.

Figure 8 – figure supplement 1: Phenotypic of effects of cell growth under A22 and cephalexin treatment. Representative phase contrast and fluorescence images of cells (CJW7323) treated with A22 (4 μg/mL) alone or with both A22 (4 μg/mL) and cephalexin (50 μg/mL) for 130 min.

Movie legends

Movie S1: Example time-lapse sequence showing ribosome and nucleoid dynamics during cell growth under steady state condition in a microfluidic channel. Shown are corresponding inverted phase contrast signal, RplA-GFP signal, HupA-mCherry signal, cell contours (based on phase contrast signals) and nucleoid contours (based on nucleoid signal segmentation) of *E. coli* cells (CJW7323) grown in a microfluidic channel supplemented with M9gluCAAT. The white circles indicate the centroid of the cell segmentation masks and the extending lines indicate the tracked cell traces.

bioRxiv preprint doi: https://doi.org/10.1101/2024.10.08.617237; this version posted March 15, 2025. The copyright holder for this preprint (which was not certified by peer review) is the author/funder, who has granted bioRxiv a license to display the preprint in perpetuity. It is made available under aCC-BY 4.0 International license.

Movie S2: Video showing the average subcellular distribution of ribosome and nucleoid signals from birth to division. Shown are 2D average RplA-GFP (top) and HupA-mCherry (bottom) projections from birth to division, with corresponding average intensity profiles (right). The cell projections are oriented from the old pole on the left to the new pole on the right. The average cell contour is also drawn. Ensemble data from 4122 division cycles of CJW7323 cells growing in M9gluCAAT are shown.

Movie S3: Video showing simulated 1D profiles of polysome and nucleoid concentration during slow and fast cell growth. Simulations during slow (left) and fast (right) growth are shown. The simulations were initialized from the equilibrium configuration, with a compact symmetric nucleoid (Dn = 10^{-3} μm^2/sec) at the cell center.

Movie S4: Video showing the effects of rifampicin addition on cell growth, ribosome signal heterogeneity, nucleoid segregation, and nucleoid compaction. Examples of five microfluidic channels showing the corresponding inverted phase contrast, RplA-GFP and HupA-mCherry signals (from left to right) of cells (CJW7323) growing within microfluidic channels. Rifampicin was added at 120 and 720 min. Each antibiotic treatment lasted 120 min.

Movie S5: Video showing the effects of ectopic polysome formation on nucleoid dynamics over time. The RplA-GFP and HupA-mCherry fluorescence normalized (norm.) by the average cell fluorescence are shown, together with their corresponding phase contrast image, for multiple cells (CJW7798) in succession following induction of mTagBFP2 expression from a T7 promoter on a multi-copy plasmid. The scale bar indicates 1 µm, and the time since cell birth is shown in minutes. The cell contours indicate the boundaries of the cell masks obtained by cell segmentation of the corresponding phase contrast images.

Movie S6 Video showing how the loss of cell width confinement due to cephalexin and A22 treatment affects ribosome and nucleoid distributions over time. Fluorescence images of RplA-GFP and HupA-mCherry fluorescence (fluor.) normalized by the average cellular fluorescence images, together with their corresponding phase contrast images, are shown for cells (CJW7323) following treatment with A22 (4 µg/mL) and cephalexin (50 µm/mL). The scale bar indicates 1 µm. The time since drug addition is shown in minutes.

bioRxiv preprint doi: https://doi.org/10.1101/2024.10.08.617237; this version posted March 15, 2025. The copyright holder for this preprint (which was not certified by peer review) is the author/funder, who has granted bioRxiv a license to display the preprint in perpetuity. It is made available under aCC-BY 4.0 International license.

Supplementary tables

Table S1. Growth medium abbreviation and composition.

Abbreviation	Composition
M9acet	1x M9 salts, 0.2% acetate (sodium salt)
M9acetCAAT	1x M9 salts, 0.2% acetate, 0.1% casamino acids, 1µg/mL thiamine
M9fuc	1x M9 salts, 0.2% fucose
M9fucCAAT	1x M9 salts, 0.2% fucose, 0.1% casamino acids, 1µg/mL thiamine
M9fum	1x M9 salts, 0.2% fumarate (disodium salt)
M9fumCAAT	1x M9 salts, 0.2% fumarate, 0.1% casamino acids, 1µg/mL thiamine
M9GlcNAc	1x M9 salts, 0.2% N-acetylglucosamine
M9GlcNAcCAAT	1x M9 salts, 0.2% N-acetylglucosamine, 0.1% casamino acids, 1µg/mL thiamine
M9glu	1x M9 salts, 0.2% glucose
M9gluCAAT	1x M9 salts, 0.2% glucose, 0.1% casamino acids, 1µg/mL thiamine
M9gly	1x M9 salts, 0.2% glycerol
M9glyCAAT	1x M9 salts, 0.2% glycerol, 0.1% casamino acids, 1µg/mL thiamine
M9Lala	1x M9 salts, 0.2% L-alanine
M9LalaCAAT	1x M9 salts, 0.2% L-alanine, 0.1% casamino acids, 1µg/mL thiamine
M9Lara	1x M9 salts, 0.2% L-arabinose
M9LaraCAAT	1x M9 salts, 0.2% L-arabinose, 0.1% casamino acids, 1µg/mL thiamine
M9mala	1x M9 salts, 0.2% malate (sodium salt)
M9malaCAAT	1x M9 salts, 0.2% malate, 0.1% casamino acids, 1µg/mL thiamine
M9malt	1x M9 salts, 0.2% maltose
M9maltCAAT	1x M9 salts, 0.2% maltose, 0.1% casamino acids, 1µg/mL thiamine
M9mann	1x M9 salts, 0.2% mannose
M9mannCAAT	1x M9 salts, 0.2% mannose, 0,1% casamino acids, 1µg/mL thiamine
M9pyr	1x M9 salts, 0.2% pyruvate (sodium salt)
M9pyrCAAT	1x M9 salts, 0.2% pyruvate, 0,1% casamino acids, 1µg/mL thiamine
M9sorb	1x M9 salts, 0.2% sorbitol
M9sorbCAAT	1x M9 salts, 0.2% sorbitol, 0,1% casamino acids, 1µg/mL thiamine
M9succ	1x M9 salts, 0.2% succinate (disodium salt)
M9succCAAT	1x M9 salts, 0.2% succinate, 0,1% casamino acids, 1µg/mL thiamine
M9treh	1x M9 salts, 0.2% trehalose
M9trehCAAT	1x M9 salts, 0.2% trehalose, 0,1% casamino acids, 1µg/mL thiamine
M9xyl	1x M9 salts, 0.2% xylose
M9xylCAAT	1x M9 salts, 0.2% xylose, 0,1% casamino acids, 1µg/mL thiamine

Table S2: *Escherichia coli* strains used in this study.

Strain	Genotype	Source
MG1655	*E. coli* MG1655 (F-lambda- *ilvG- rfb-50 rph-1*)	(Guyer et al., 1981; Jensen, 1993)
MG1655 (DE3)	*E. coli* MG1655 (DE3)	Kind gift from Dr K. Prather (Massachusetts Institute of Technology), (Tseng et al., 2010b)
CJW5158	*E. coli* BW25113 *hupA::hupA-mcherry-frt-kanR-frt*	(Gray et al., 2019)
CJW6723	*E. coli* MG1655 Δ*lacZYA*::P_{lac}*-egfp-µNS hupA:hupA-mcherry*	(Gray et al., 2019)
CJW6768	*E. coli* MG1655 *rplA::rplA-meos2*	(Sanamrad et al., 2014)
CJW6769	*E. coli* MG1655 *rspB::rpsB-meos2*	(Sanamrad et al., 2014)
CJW7019	*E. coli* MG1655 *rplA::rplA-msfgfp-frt-kanR-frt*	(Gray et al., 2019)
CJW7020	*E. coli* MG1655 *rplA::rplA-msfgfp*	(Gray et al., 2019)
CJW7021	*E. coli* MG1655 *rpsB::rpsB-msfgfp*	(Gray et al., 2019)
CJW7144	*E. coli* MG1655 Δ*lacZYA*::P_{lac}*-mcherry-µNS-frt-kanR-frt*	This study
CJW7145	*E. coli* MG1655 Δ*lacZYA*::P_{lac}*-mcherry-µNS-frt-kanR-frt rplA::rplA-msfgfp*	This study
CJW7323	*E. coli* MG1655 *rplA::rplA-msfgfp hupA::hupA-mcherry*	(Xiang et al., 2021)
CJW7466	*E. coli* MG1655 (DE3) *hupA:hupA-mcherry*	This study
CJW7651	*E. coli* MG1655 Δ*lacZYA*::P_{lac}*-mcherry-µNS rplA::rplA-msfgfp*	This study
CJW7766	*E. coli* MG1655 (DE3) *hupA:hupA-mcherry rplA::rplA-msfgfp-frt-kanR-frt*	This study
CJW7798	*E. coli* MG1655 (DE3) *hupA:hupA-mcherry rplA::rplA-msfgfp-frt-kanR-frt* | *pET28:mTagBFP2-CmR*	This study

bioRxiv preprint doi: https://doi.org/10.1101/2024.10.08.617237; this version posted March 15, 2025. The copyright holder for this preprint (which was not certified by peer review) is the author/funder, who has granted bioRxiv a license to display the preprint in perpetuity. It is made available under aCC-BY 4.0 International license.

Table S3: Plasmids used in this study.

Plasmid name	Relevant genetic elements	Source
pKD13	*frt-kanR-frt-R6Kori-ampR*	(Datsenko and Wanner, 2000)
pKD46	*araC-bet-exo-A101(Ts)ori-ampR*	(Datsenko and Wanner, 2000)
pCP20	*cmR-A101(Ts)ori-ampR-flp-λ-repressor(Ts)*	(Datsenko and Wanner, 2000)
pER12	*pBAD322A-gfp-μNS*	Kind gift from Dr A. Janakiraman (City College of New York), (Broering et al., 2005)
pER12-mcherry	*pBAD322A-mcherry-μNS*	This study
pAPG1	*attB-pBAD322A-mcherry-μNS-attB-kanR-ColE1ori*	This study
pSB3C5-proA-B0032-E0051	*PproA-lacZα-GFP-p15Aori-cmR*	(Davis et al., 2011)
pET28:GFP	*PT7-lacO-GFP-kanR-pMB1ori-lacI*	(Shis and Bennett, 2013)
pBAD:TagBFP2	*araC-ParaBAD-mTagBFP2-ampR-pBAD322ori*	(Subach et al., 2011)
pET28:mTagBFP2	*PT7-lacO-mTagBFP2-kanR-pMB1ori-lacI*	This study
pET28:mTagBFP2-CmR	*PT7-lacO-mTagBFP2-cmR-pMB1ori-lacI*	This study

Table S4: DNA oligonucleotides used in this study.

Oligo name	Sequence	Source
ER12-MCR-fwd	5' CTCCATACCCGTTTTTTTGGGCTAGCAGGAGGAATTCATGGTGAGCAAGGGCGAGGAG 3'	This study
ER12-MCR-rev2	5' CACTGGAGGAGCCTGCTTTTTTGTACAAACTTGTTGACTTGTACAGCTCGTCCATG 3'	This study
μNSmCherry fwd	5' CACAGGTTGCTCCGGGCTATGAAATAGAAAAATGAATCCGTTGAAGCCTGATCGATGCATAATGTGCC 3'	This study
μNSmCherry rev	5' AGCTCCAGCCTACACAGAGTTTGTAGAAACGCAAAAAG 3'	This study
FRT_KanR fwd	5' GTTTCTACAAACTCTGTGTAGGCTGGAGCTGC 3'	This study
FRT_KanR Rev	5' TTAAAGGTATTAAAAACAACTTTTTGTCTTTTTACCTTCCCGTTTCGCTCCTGTCAAACATGAGAATTAATTCC 3'	This study
ColE1 fwd	5' GAGCGAAACGGGAAGGTAAAAAGACAAAAAGTTGTTTTTAATACCTTTAACGTTCCACTGAGCGTC 3'	This study
ColE1 rev	5' CAGGCTTCAACGGATTCATTTTTCTATTTCATAGCCCGGAGCAACCTGTGGGTTATCCACAGAATCAGG 3'	This study
pAPG1 seq1	5' GATCAAGCAGAGGCTGAAG 3'	This study
pAPG1 seq2	5' CCAGGCATCAAATTAAGC 3'	This study
pAPG1 seq3	5' TCTACGTGTTCCGCTTCC 3'	This study
pAPG1 seq4	5' TTGAAGCCTGATCGATGC 3'	This study
pAPG1 seq5	5' AAGATTAGCGGATCCTACC 3'	This study
lacZYA_redμNS fwd	5' TATGTTGTGTGGAATTGTGAGCGGATAACAATTTCACACAGGAAACAGCTATGGTGAGCAAGGGCGA 3'	This study
lacZYA_redμNS rev	5' CAATTTTTATAATTTAAACTGACGATTCAACTTTATAATCTTTGAAATAAGGATCCGTCGACCTGCAG 3'	This study
LacI fwd	5' GGCCGATTCATTAATGCAGCTGGC 3'	This study
CynX rev	5' GGCCTGATAAGCGCAGCGTATC 3'	This study
mCherry rev	5' GGTGCTTCACGTAGGCCTTGG 3'	This study
KanR fwd	5' CGGAGAACCTGCGTGCAATCC 3'	This study
mTagBFP2 fwd	5' CGGAGCTCGAATTCGGATCCTTAATTAAGCTTGTGCCCCAGTTTG 3'	This study
mTagBFP2 fwd	5' CTTTAAGAAGGAGATATACCATGAGCGAGCTGATTAAGGAGAAC 3'	This study
pET28_one fwd	5' TCCTTAATCAGCTCGCTCATGGTATATCTCCTTCTTAAAGTTAAAC 3'	This study
pET28_one rev	5' GGATAACCGTATTACCGCCTTTGAGTGAGCTGATACCG 3'	This study
pET28_two fwd	5' AGCGGTATCAGCTCACTCAAAGGCGGTAATACGGTTATCC 3'	This study
pET28_two rev	5' TGGGGCACAAGCTTAATTAAGGATCCGAATTCGAGCTCC 3'	This study
pET28mTagBFP2 fwd	5' CTTAGTGACTCGAATTCGCGCGCCAATCCGGATATAGTTCC 3'	This study
pET28mTagBFP2 rev	5' ACTTTCTGGCTGGATGATGGACGTGAGTTTTCGTTCCACTG 3'	This study
cmR fwd	5' AGTGGAACGAAAACTCACGTCCATCATCCAGCCAGAAAGTG 3'	This study
cmR rev	5' GAACTATATCCGGATTGGCGCGCGAATTCGAGTCACTAAGG 3'	This study

bioRxiv preprint doi: https://doi.org/10.1101/2024.10.08.617237; this version posted March 15, 2025. The copyright holder for this preprint (which was not certified by peer review) is the author/funder, who has granted bioRxiv a license to display the preprint in perpetuity. It is made available under aCC-BY 4.0 International license.

Table S5: Chemicals used in this study.

Chemical	Source	Catalog number
Agarose	AmericanBio	Cat#AB00972-00500
4',6-diamidine-2′-phenylindole (DAPI), dihydrochloride fluorescent dye	Thermo Fisher Scientific	Cat#D1306
Isopropyl β-D-1-thiogalactopyranoside (IPTG)	Sigma Aldrich	Cat#I5502
Rifampicin	Sigma Aldrich	Cat#R3501
Cephalexin	Sigma Aldrich	Cat#C4895
A22	Sigma Aldrich	Cat#SML0471
**Chloramphenicol**	Sigma Aldrich	Cat#C0378
4',6-Diamidine-2-phenylindole dihydrochloride (DAPI)	Thermo Fisher Scientific	Cat#D1306
eBioscience™ DRAQ5™	Thermo Fisher Scientific	Cat#65-0880-92

Table S6: Software used in this study.

Program / library / script	Package	Source
Oufti	www.oufti.org	(Paintdakhi et al., 2016)
MATLAB	www.mathworks.com	Mathworks
Python	www.python.org	Python Software Foundation
Numpy	www.numpy.org	(Harris et al., 2020)
Scipy	www.scipy.org	(Virtanen et al., 2020)
Pytorch	www.pytorch.org	(Paszke et al., 2019)
Scikit-image	www.scikit-image.org	(Van Der Walt et al., 2014)
Scikit-learn	www.scikit-learn.org/stable/	(Pedregosa et al., 2012)
Statsmodels	www.statsmodels.org/stable/index.html	(Seabold and Perktold, 2010)
Shapely	www.pypi.org/project/shapely/	(Gillies, Sean et al., 2023)
Matplotlib	www.matplotlib.org	(Hunter, 2007)
Seaborn	www.seaborn.pydata.org	(Waskom, 2021)
Pandas	www.pandas.pydata.org	(McKinney, 2010)
Python	Omnipose neural network	(Cutler et al., 2022)
MATLAB	SuperSegger	(Stylianidou et al., 2016)
Python	Unet neural network	(Wiktor et al., 2021; Zhou et al., 2020)
snapshots_analysis_UNET_ghv.py	Snapshot image analysis (from UNET masks) – custom Python class	This study (www.github.com/JacobsWagnerLab/published/tree/master/Papagiannakis_2025)
snapshots_analysis_OUFTI_GrayGovers_ghv.py	Snapshot image analysis (from Oufti masks) – custom Python class	This study (www.github.com/JacobsWagnerLab/published/tree/master/Papagiannakis_2025)
snapshots_analysis_functions.py	Extraction of fluorescence and morphology statistics from cell snapshots – custom Python functions	This study (www.github.com/JacobsWagnerLab/published/tree/master/Papagiannakis_2025)
microfluidics_segmentation_ghv.py	Cell segmentation and tracking from time-lapse images in microfluidics – custom Python class	This study (www.github.com/JacobsWagnerLab/published/tree/master/Papagiannakis_2025)
microfluidics_analysis_functions_ghv.py	Extraction of fluorescence and morphology statistics from time-lapse images	This study (www.github.com/JacobsWagnerLab/published/tree/master/Papagiannakis_2025)

bioRxiv preprint doi: https://doi.org/10.1101/2024.10.08.617237; this version posted March 15, 2025. The copyright holder for this preprint (which was not certified by peer review) is the author/funder, who has granted bioRxiv a license to display the preprint in perpetuity. It is made available under aCC-BY 4.0 International license.

	in microfluidics – custom Python functions	
Omnipose_to_python_ghv.py	Extraction of segmentation masks and tracked lineages from Omnipose/SuperSegger to Python for further analysis	This study (www.github.com/JacobsWagnerLab/published/tree/master/Papagiannakis_2025)
Otsu_phase_segmentation_ghv.py	Cell segmentation and tracking using Otsu thresholding (Otsu, 1979)	This study (www.github.com/JacobsWagnerLab/published/tree/master/Papagiannakis_2025)
LoG_adaptive_image_filter.py	Image filter that applies relative and local thresholding to segment fluorescence objects and particles	This study (www.github.com/JacobsWagnerLab/published/tree/master/Papagiannakis_2025)
Microfluidics_segmentation_and_tracking_example.pdf	Example for cell segmentation and tracking in microfluidics	This study (www.github.com/JacobsWagnerLab/published/tree/master/Papagiannakis_2025)
Analysis_of_cell_morphology_and_fluorescence_in_microfluidics.pdf	Example for the extraction of fluorescence and morphology statistics from time-lapse images in microfluidics	This study (www.github.com/JacobsWagnerLab/published/tree/master/Papagiannakis_2025)
Analysis_of_cell_morphology_and_fluorescence_in_agarose_pads.pdf	Example for the extraction of fluorescence and morphology statistics from cell snapshots	This study (www.github.com/JacobsWagnerLab/published/tree/master/Papagiannakis_2025)
Example_of_using_Otsu_based_segmentation_ghv.pdf	Example for the segmentation of antibiotic-treated cells using the Otsu threshold	This study (www.github.com/JacobsWagnerLab/published/tree/master/Papagiannakis_2025)
tune_diffusion.ipynb	Simulations of the minimal reaction-diffusion model that describes nucleoid segregation in *E. coli*.	This study (https://github.com/qiweiyuu/polysome)
Time_lapse_on_agarose_pad	Python repository that includes the *fluorescence_analysis* class and its associated functions, used to track cell trajectories and analyze their fluorescence statistics in time-lapse images on agarose pads.	This study (https://github.com/alexSysBio/Time_lapse_on_agarose_pad)
flowio_to_pandas	Python repository that includes the *flow_cytometry_class* class and its associated functions, used to parse FCS files into Pandas. This class can also be used for polygon and histogram gating.	This study (https://github.com/alexSysBio/flowio_to_pandas)
nd2_to_array.py	Function used to	This study

bioRxiv preprint doi: https://doi.org/10.1101/2024.10.08.617237; this version posted March 15, 2025. The copyright holder for this preprint (which was not certified by peer review) is the author/funder, who has granted bioRxiv a license to display the preprint in perpetuity. It is made available under aCC-BY 4.0 International license.

	parse .nd2 files into numpy arrays.	(https://github.com/alexSysBio/Adding_ND2_images_to_python)
Bivariate_medial_axis_estimation.py	Functions used to draw the medial axis in segmented cell masks from snapshots or time-lapse images on agarose pads.	This study (https://github.com/alexSysBio/Cell_medial_axis_definitions)
Unever_background_correction.py	Functions used to subtract the background from agarose-pad images.	This study (https://github.com/alexSysBio/Image_background_subtraction)

2549

bioRxiv preprint doi: https://doi.org/10.1101/2024.10.08.617237; this version posted March 15, 2025. The copyright holder for this preprint (which was not certified by peer review) is the author/funder, who has granted bioRxiv a license to display the preprint in perpetuity. It is made available under aCC-BY 4.0 International license.

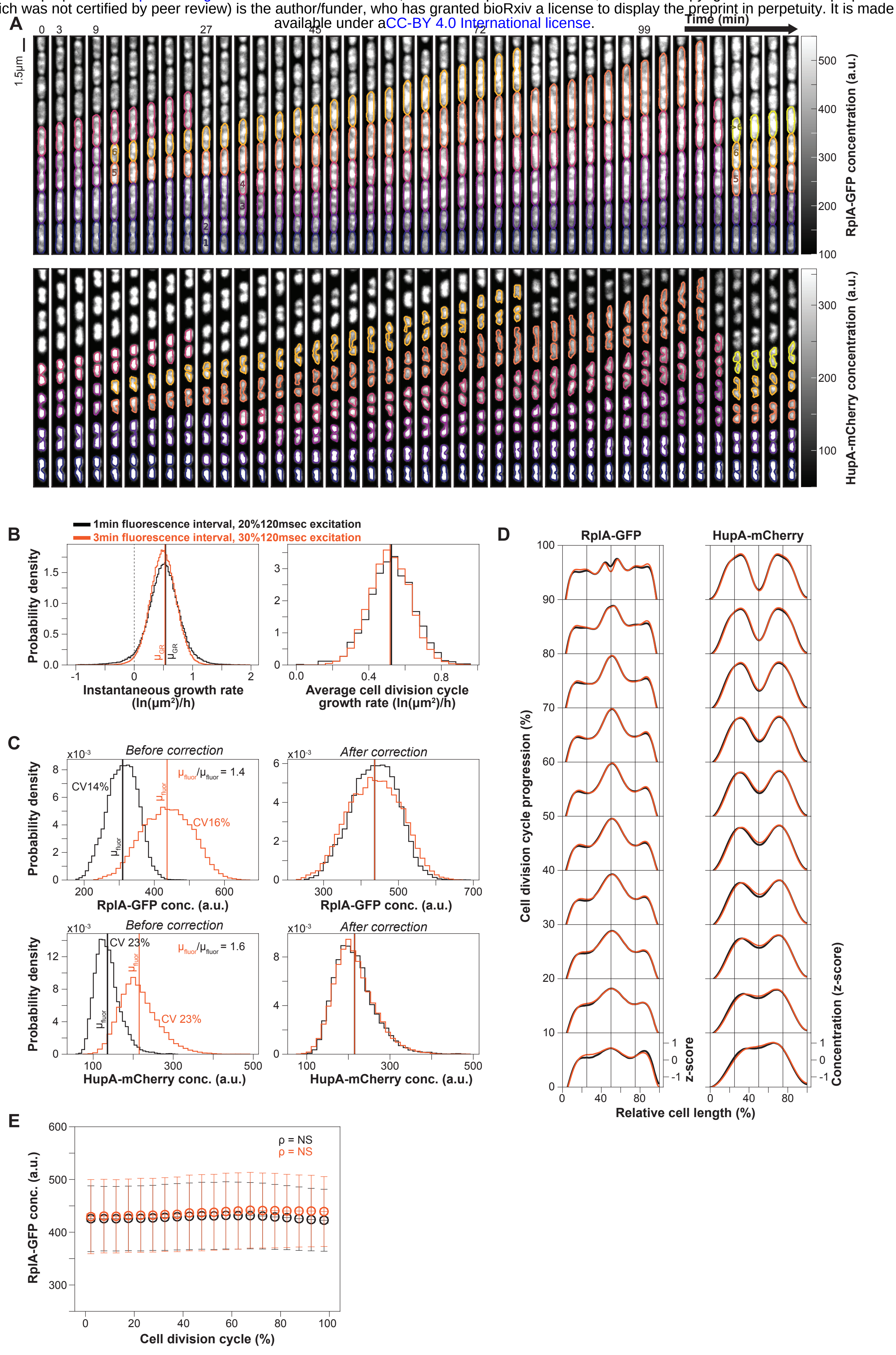

Figure 1 - figure supplement 1

bioRxiv preprint doi: https://doi.org/10.1101/2024.10.08.617237; this version posted March 15, 2025. The copyright holder for this preprint (which was not certified by peer review) is the author/funder, who has granted bioRxiv a license to display the preprint in perpetuity. It is made available under aCC-BY 4.0 International license.

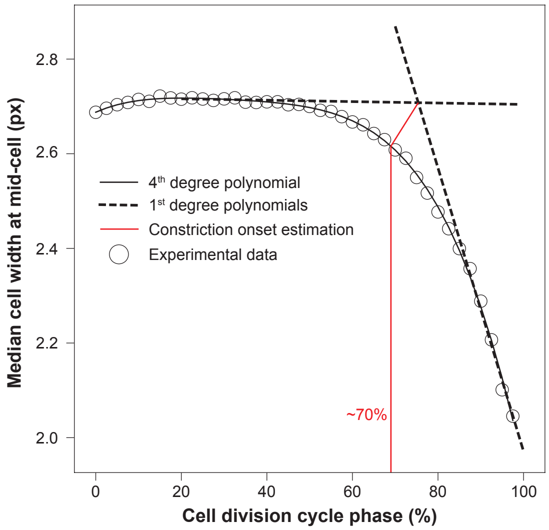

Figure 1 - Figure supplement 2

bioRxiv preprint doi: https://doi.org/10.1101/2024.10.08.617237; this version posted March 15, 2025. The copyright holder for this preprint (which was not certified by peer review) is the author/funder, who has granted bioRxiv a license to display the preprint in perpetuity. It is made available under aCC-BY 4.0 International license.

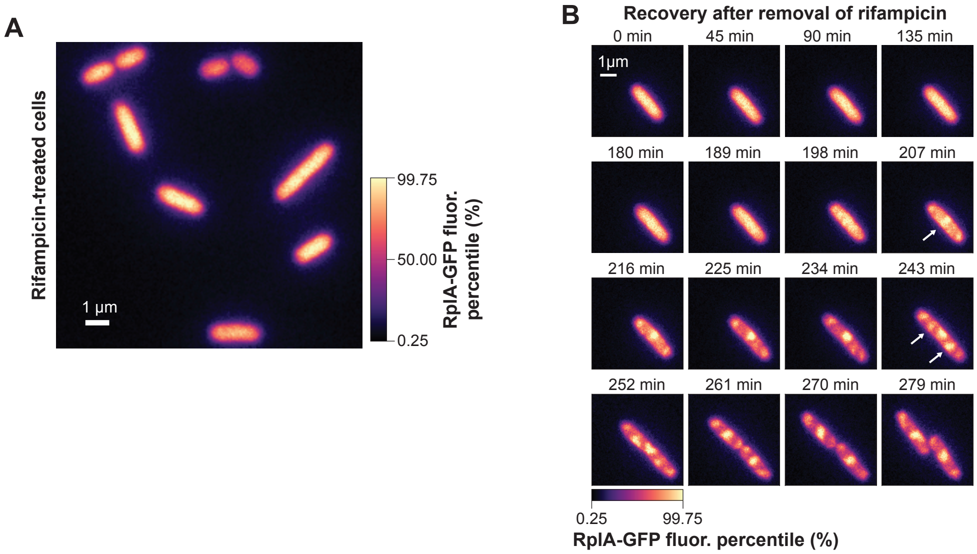

Figure 1 - figure supplement 3

bioRxiv preprint doi: https://doi.org/10.1101/2024.10.08.617237; this version posted March 15, 2025. The copyright holder for this preprint (which was not certified by peer review) is the author/funder, who has granted bioRxiv a license to display the preprint in perpetuity. It is made available under aCC-BY 4.0 International license.

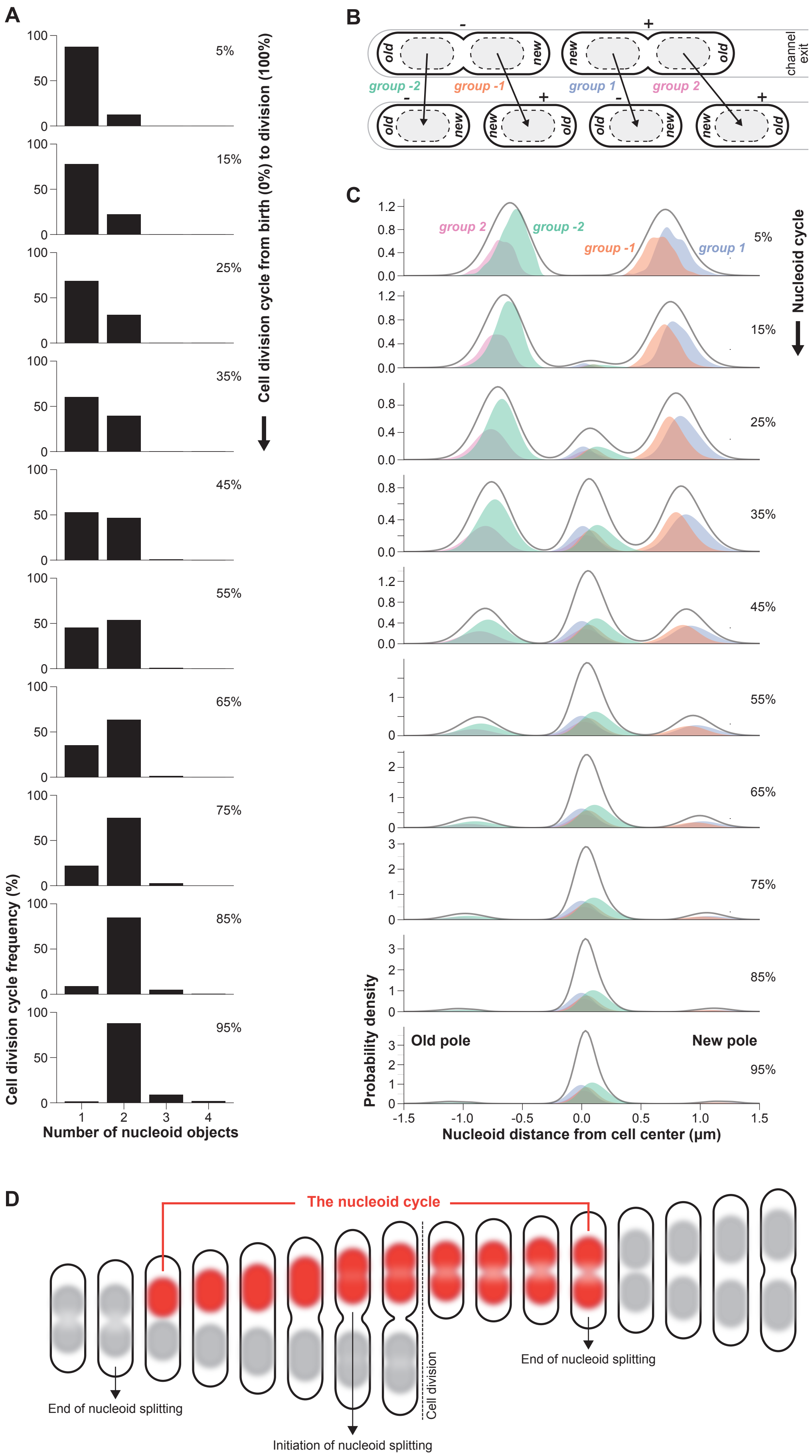

Figure 1 - figure supplement 4

bioRxiv preprint doi: https://doi.org/10.1101/2024.10.08.617237; this version posted March 15, 2025. The copyright holder for this preprint (which was not certified by peer review) is the author/funder, who has granted bioRxiv a license to display the preprint in perpetuity. It is made available under aCC-BY 4.0 International license.

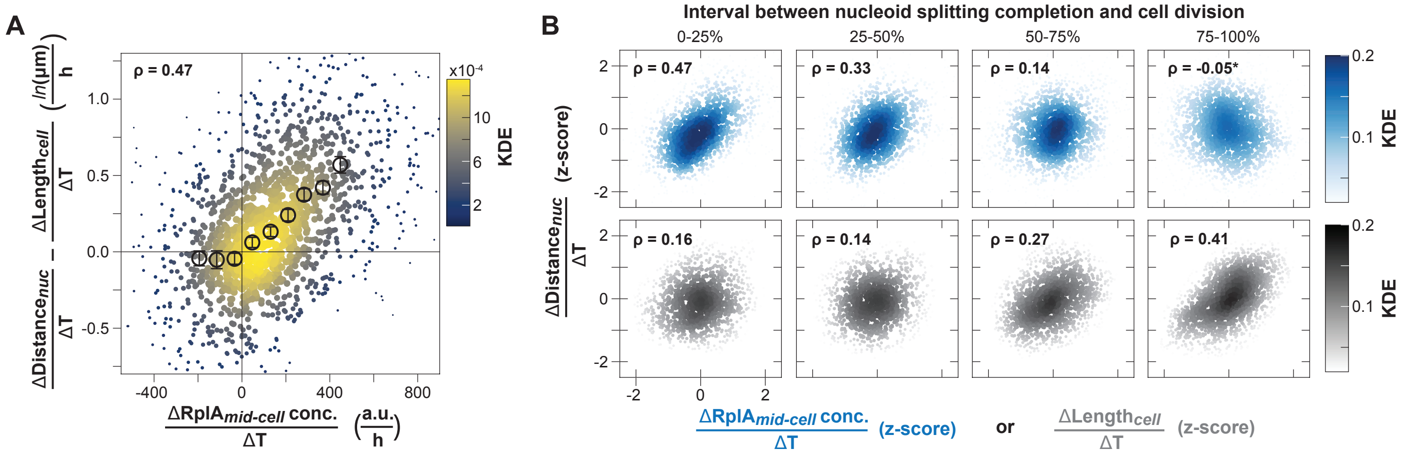

Figure 1 - figure supplement 5

bioRxiv preprint doi: https://doi.org/10.1101/2024.10.08.617237; this version posted March 15, 2025. The copyright holder for this preprint (which was not certified by peer review) is the author/funder, who has granted bioRxiv a license to display the preprint in perpetuity. It is made available under aCC-BY 4.0 International license.

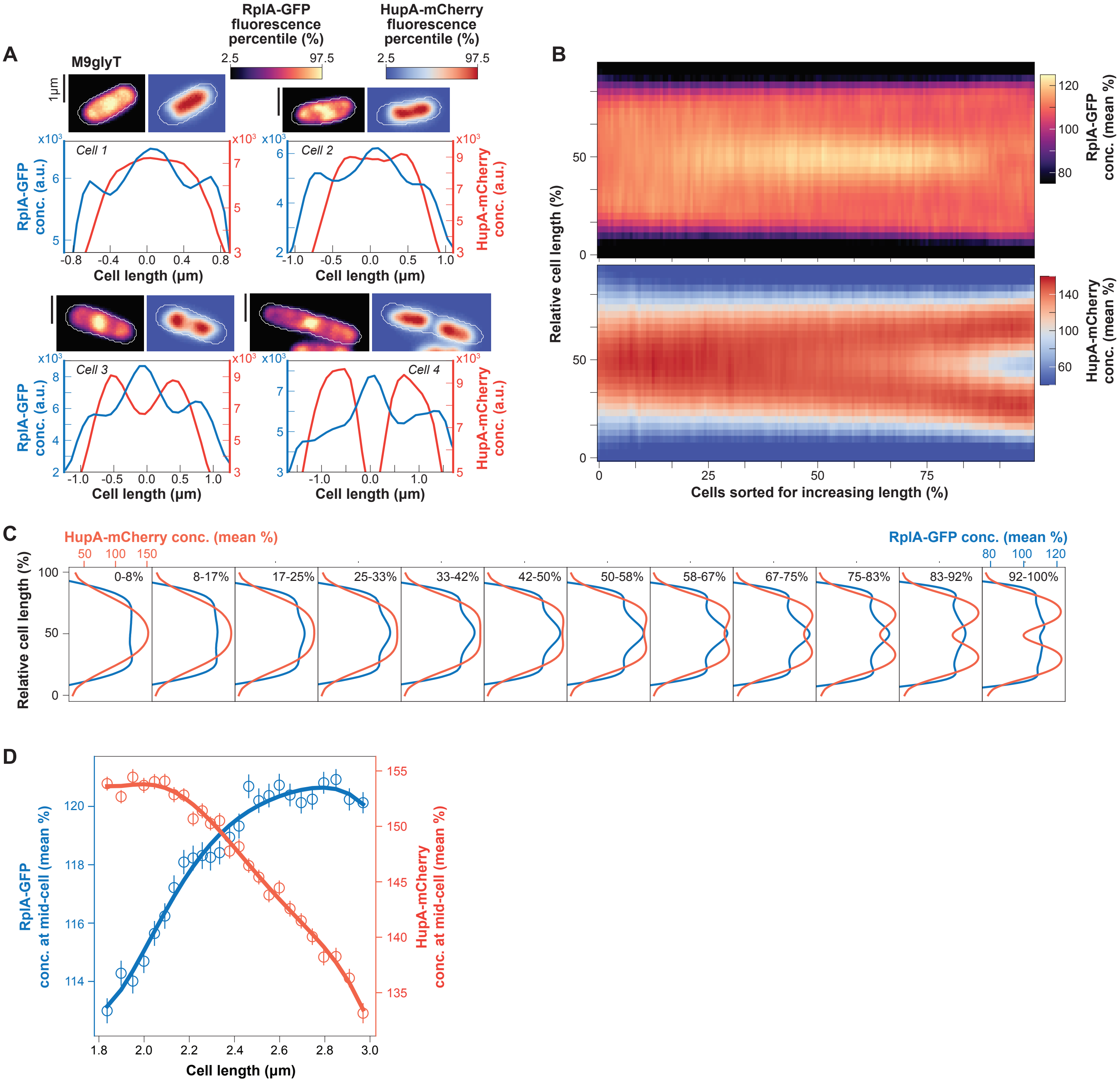

Figure 1 - figure supplement 6

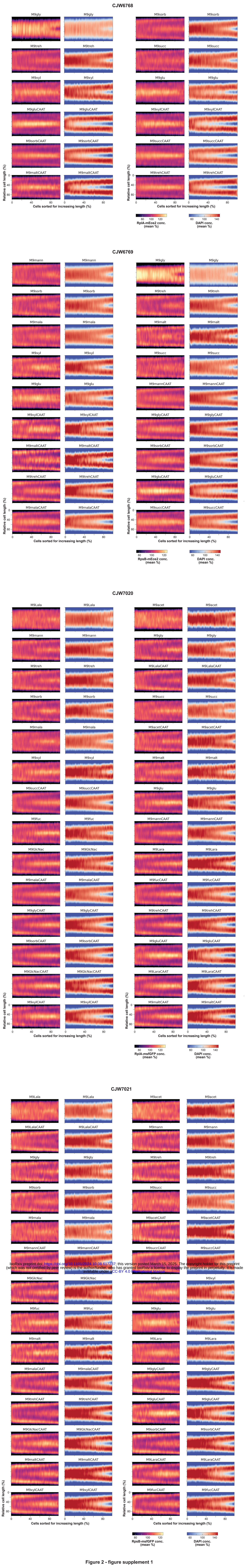

Figure 2 - figure supplement 1

bioRxiv preprint doi: https://doi.org/10.1101/2024.10.08.617237; this version posted March 15, 2025. The copyright holder for this preprint (which was not certified by peer review) is the author/funder, who has granted bioRxiv a license to display the preprint in perpetuity. It is made available under aCC-BY 4.0 International license.

bioRxiv preprint doi: https://doi.org/10.1101/2024.10.08.617237; this version posted March 15, 2025. The copyright holder for this preprint (which was not certified by peer review) is the author/funder, who has granted bioRxiv a license to display the preprint in perpetuity. It is made available under aCC-BY 4.0 International license.

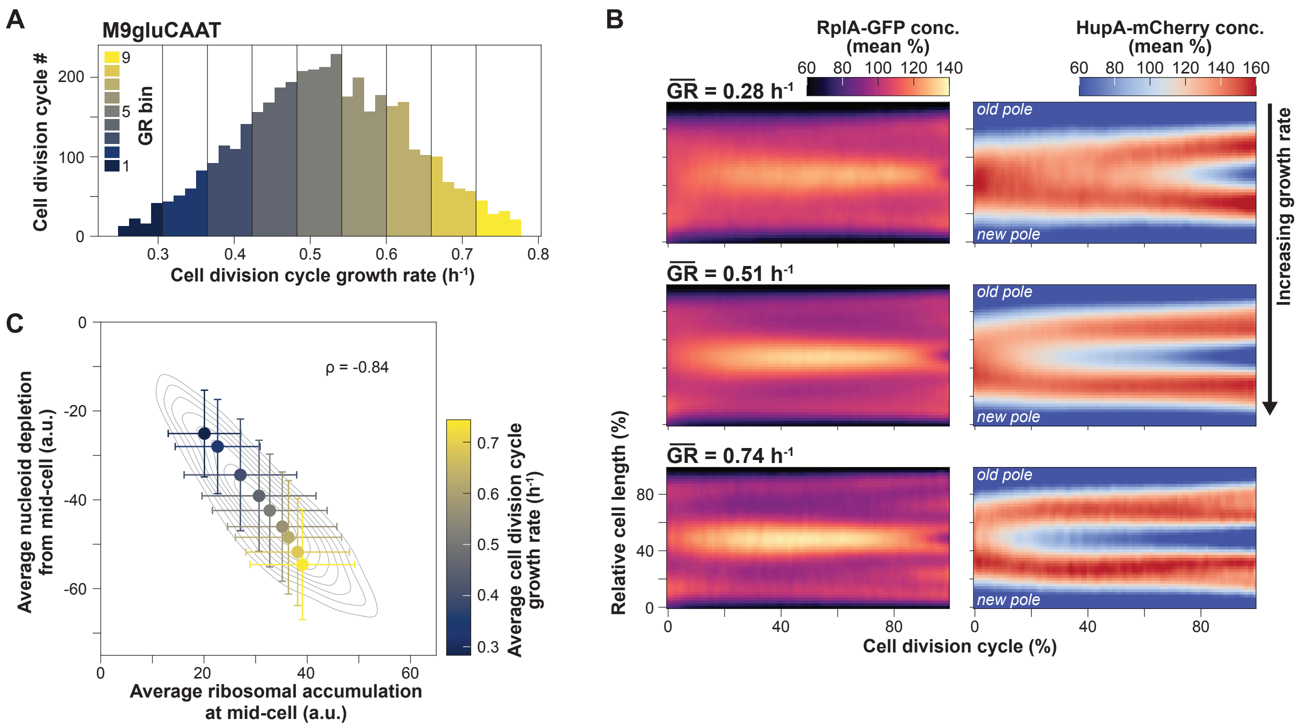

Figure 2 - figure supplement 2

bioRxiv preprint doi: https://doi.org/10.1101/2024.10.08.617237; this version posted March 15, 2025. The copyright holder for this preprint (which was not certified by peer review) is the author/funder, who has granted bioRxiv a license to display the preprint in perpetuity. It is made available under aCC-BY 4.0 International license.

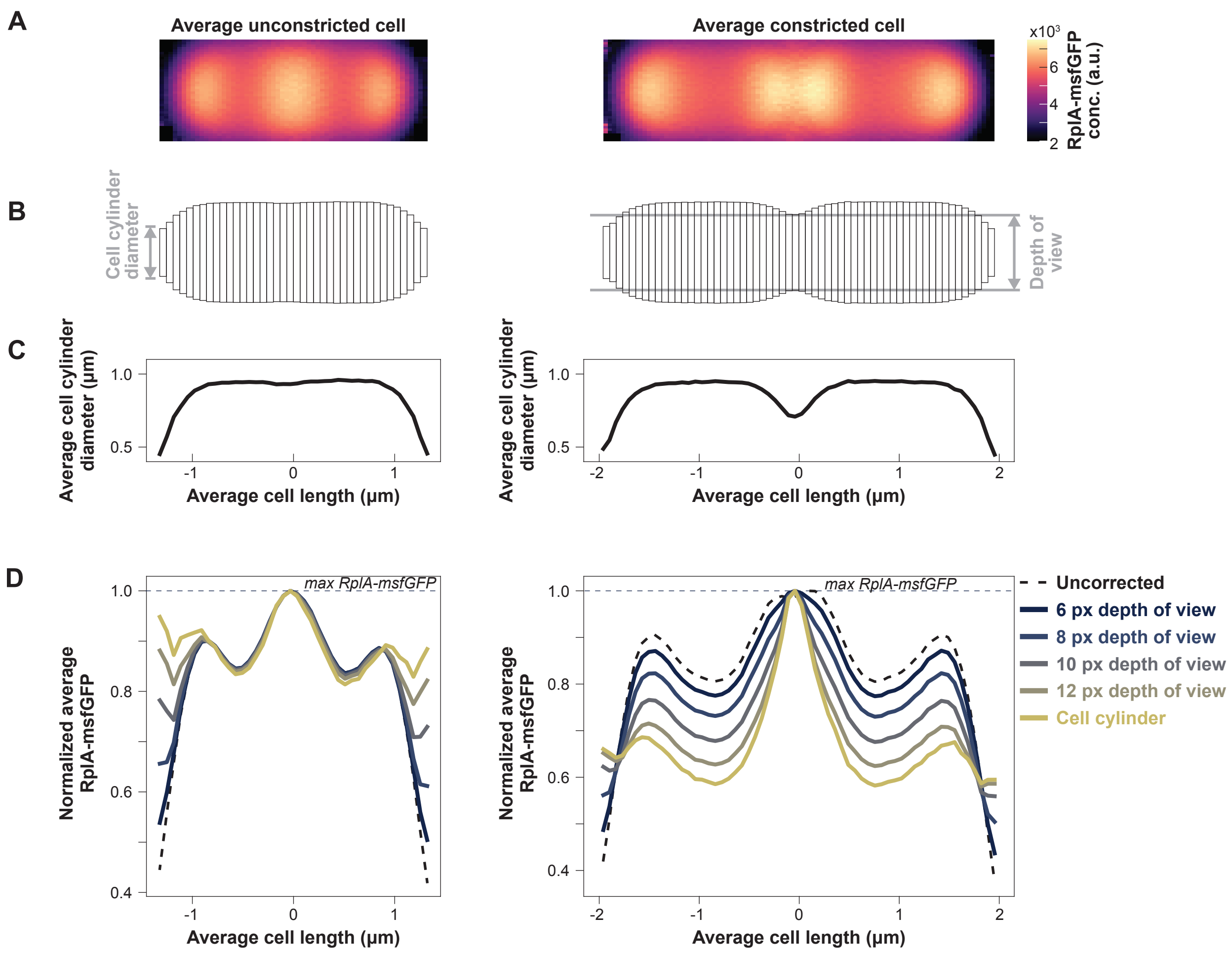

Figure 2 - figure supplement 3

bioRxiv preprint doi: https://doi.org/10.1101/2024.10.08.617237; this version posted March 15, 2025. The copyright holder for this preprint (which was not certified by peer review) is the author/funder, who has granted bioRxiv a license to display the preprint in perpetuity. It is made available under aCC-BY 4.0 International license.

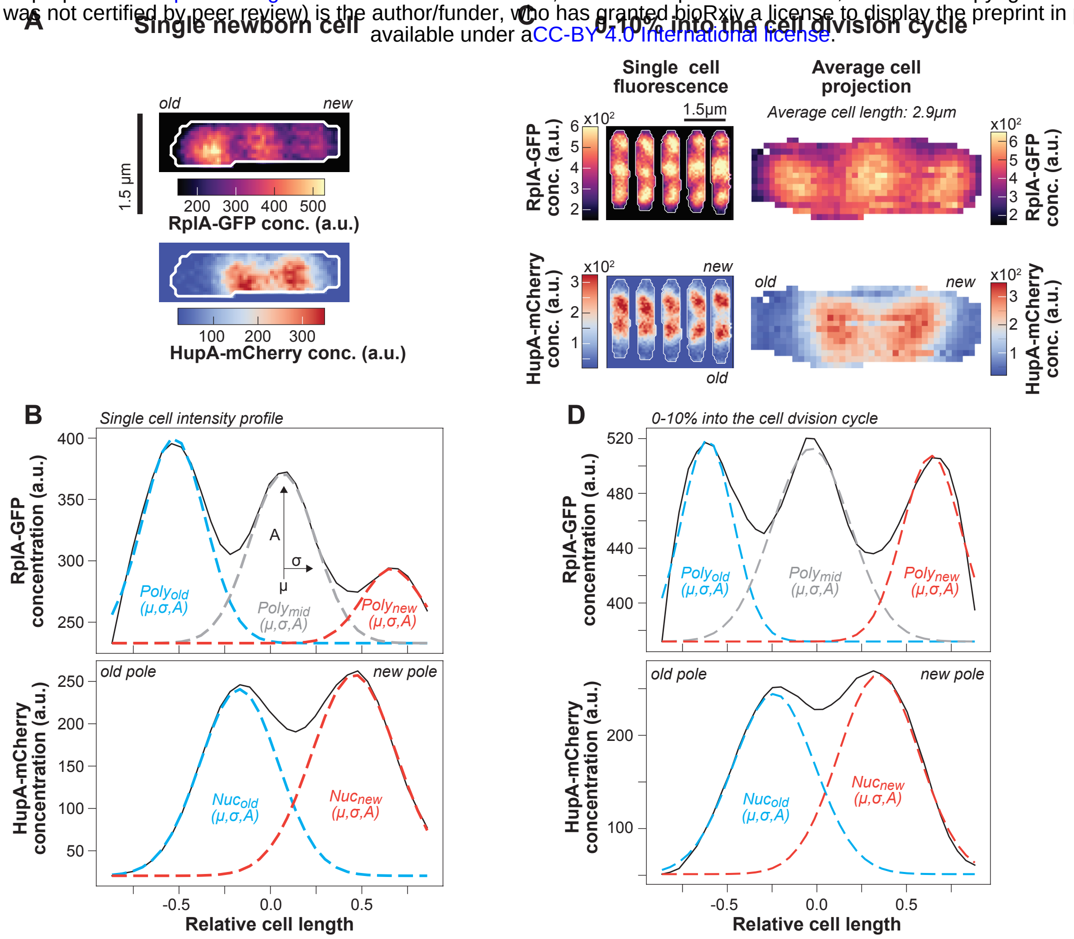

Figure 3 - figure supplement 1

bioRxiv preprint doi: https://doi.org/10.1101/2024.10.08.617237; this version posted March 15, 2025. The copyright holder for this preprint (which was not certified by peer review) is the author/funder, who has granted bioRxiv a license to display the preprint in perpetuity. It is made available under aCC-BY 4.0 International license.

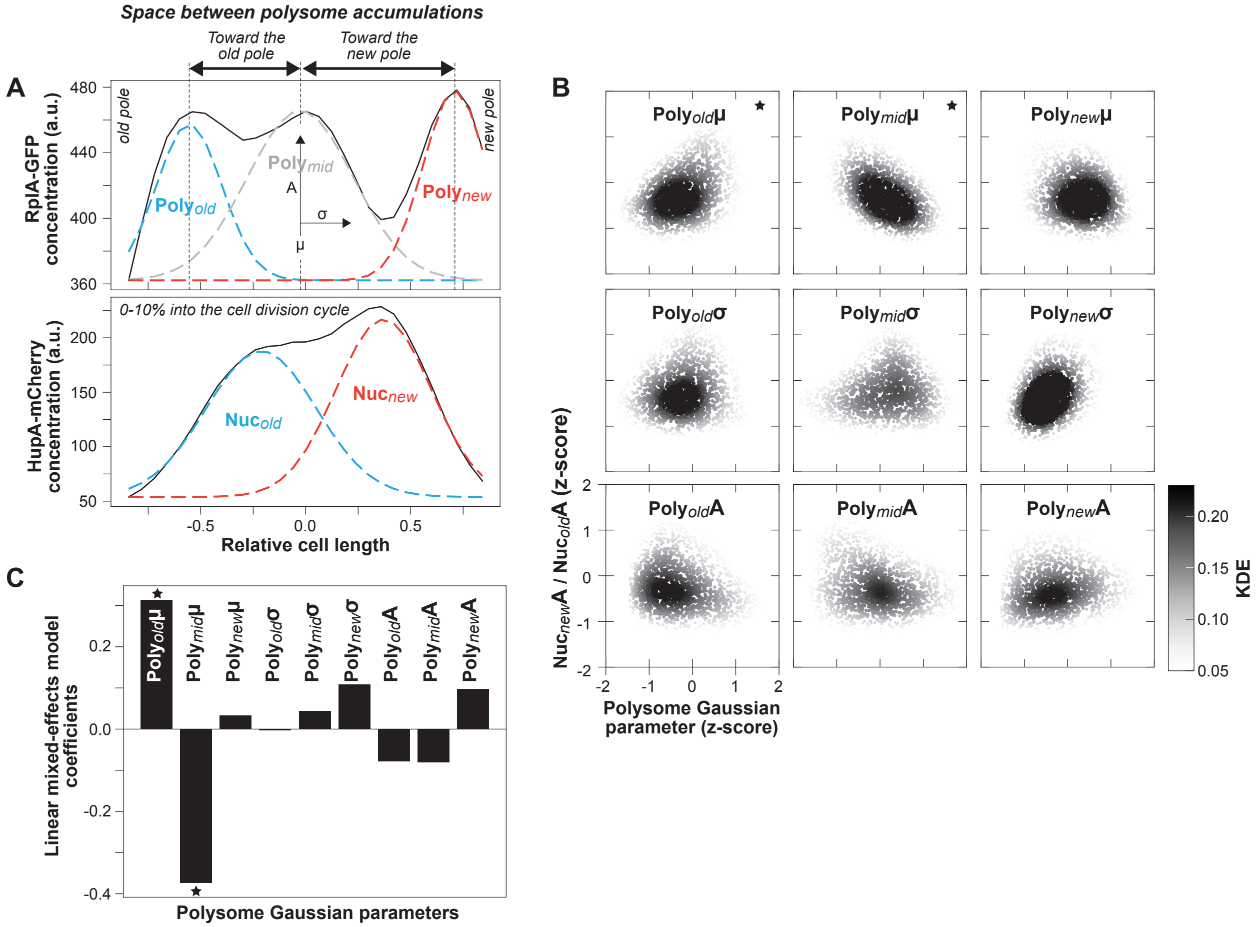

Figure 3 - figure supplement 2

bioRxiv preprint doi: https://doi.org/10.1101/2024.10.08.617237; this version posted March 15, 2025. The copyright holder for this preprint (which was not certified by peer review) is the author/funder, who has granted bioRxiv a license to display the preprint in perpetuity. It is made available under aCC-BY 4.0 International license.

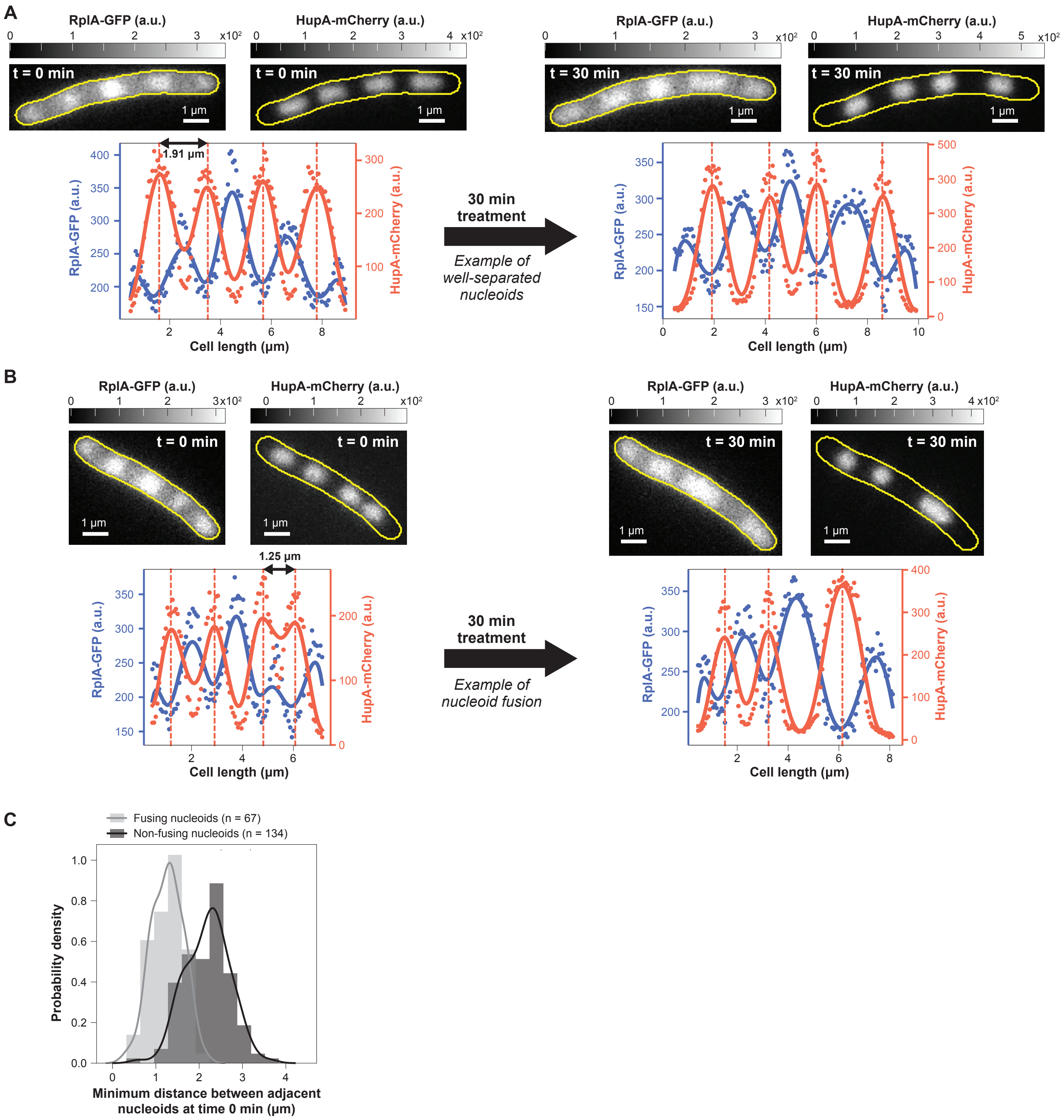

Figure 4 - Figure supplement 1

bioRxiv preprint doi: https://doi.org/10.1101/2024.10.08.617237; this version posted March 15, 2025. The copyright holder for this preprint (which was not certified by peer review) is the author/funder, who has granted bioRxiv a license to display the preprint in perpetuity. It is made available under aCC-BY 4.0 International license.

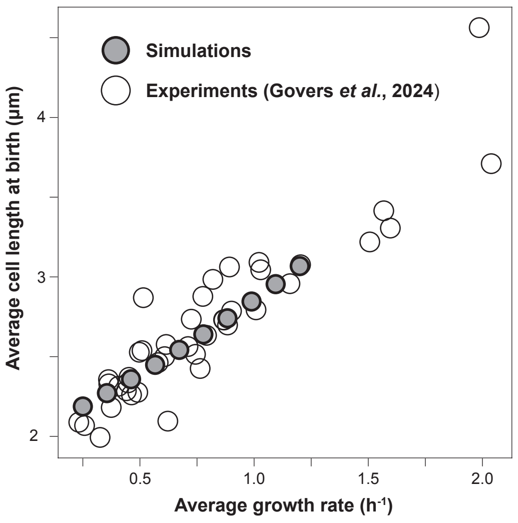

Figure 4 - figure supplement 2

bioRxiv preprint doi: https://doi.org/10.1101/2024.10.08.617237; this version posted March 15, 2025. The copyright holder for this preprint (which was not certified by peer review) is the author/funder, who has granted bioRxiv a license to display the preprint in perpetuity. It is made available under aCC-BY 4.0 International license.

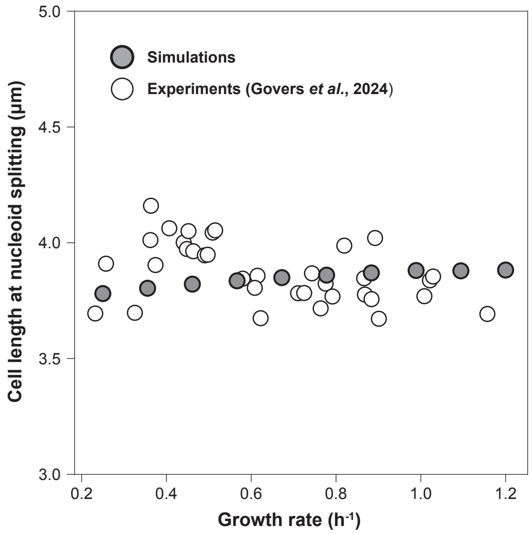

Figure 4 - figure supplement 3

bioRxiv preprint doi: https://doi.org/10.1101/2024.10.08.617237; this version posted March 15, 2025. The copyright holder for this preprint (which was not certified by peer review) is the author/funder, who has granted bioRxiv a license to display the preprint in perpetuity. It is made available under aCC-BY 4.0 International license.

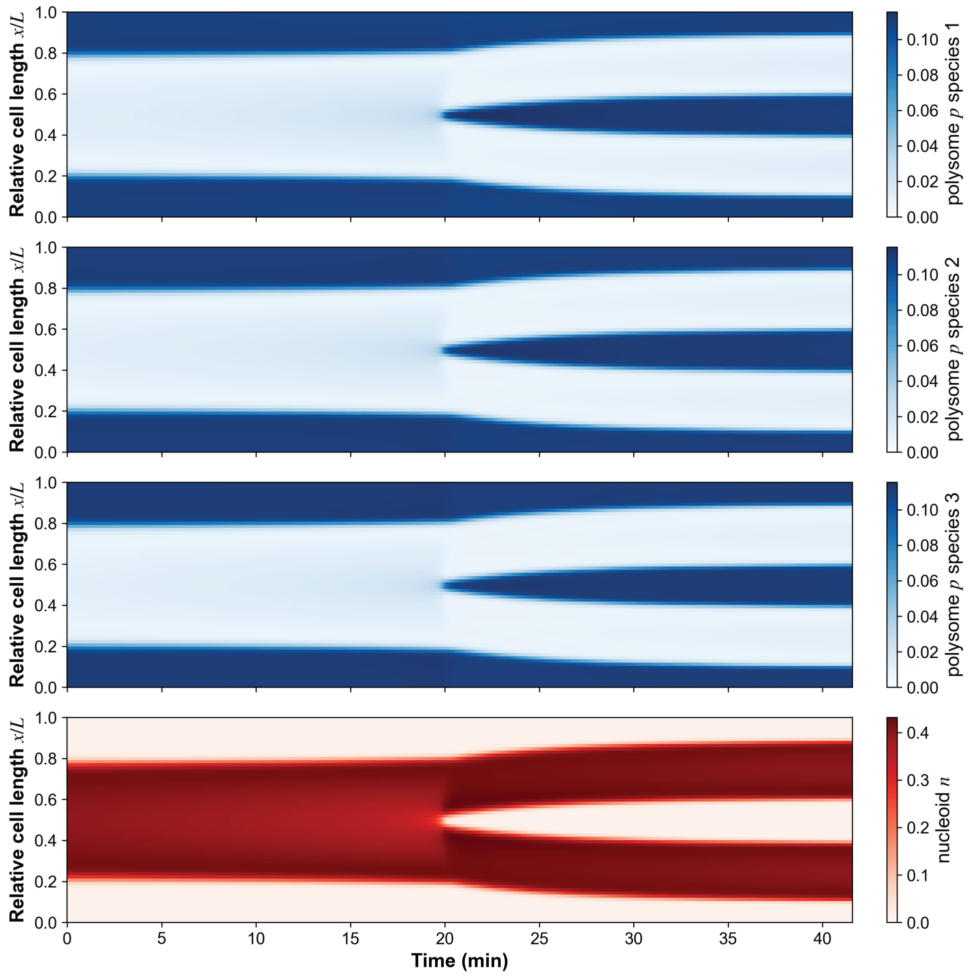

Figure 4 - figure supplement 4

bioRxiv preprint doi: https://doi.org/10.1101/2024.10.08.617237; this version posted March 15, 2025. The copyright holder for this preprint (which was not certified by peer review) is the author/funder, who has granted bioRxiv a license to display the preprint in perpetuity. It is made available under aCC-BY 4.0 International license.

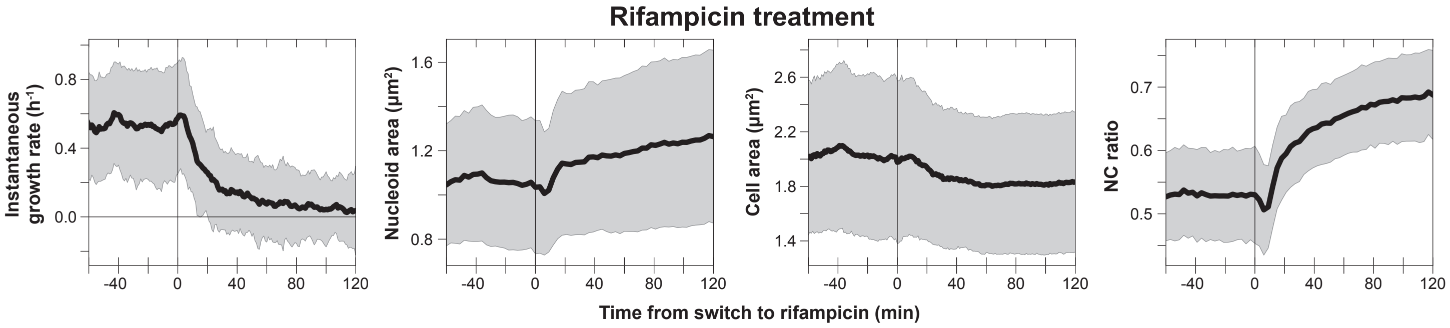

Figure 6 - figure supplement 1

bioRxiv preprint doi: https://doi.org/10.1101/2024.10.08.617237; this version posted March 15, 2025. The copyright holder for this preprint (which was not certified by peer review) is the author/funder, who has granted bioRxiv a license to display the preprint in perpetuity. It is made available under aCC-BY 4.0 International license.

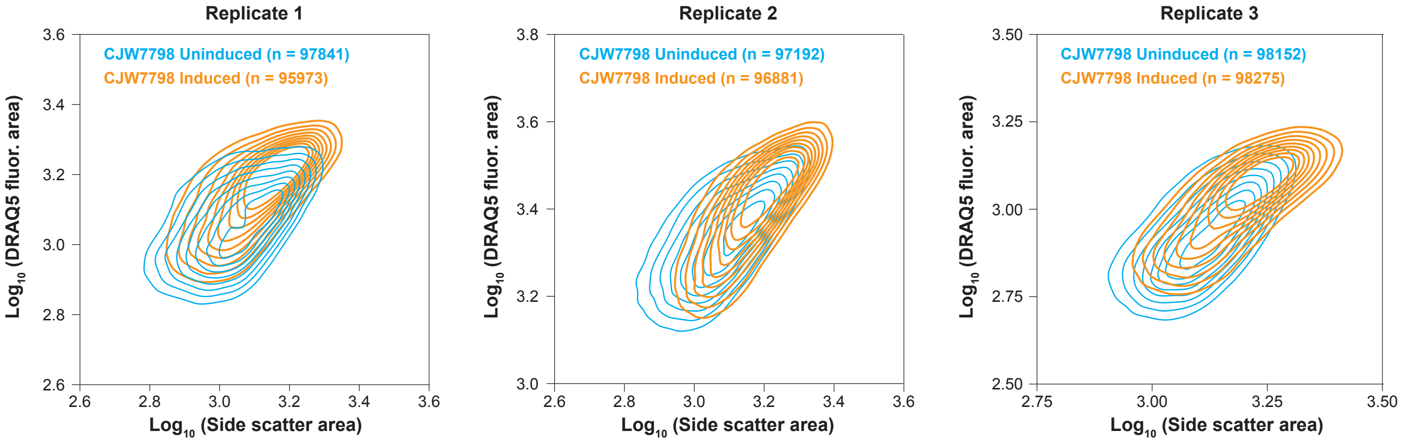

Figure 7 - figure supplement 1

bioRxiv preprint doi: https://doi.org/10.1101/2024.10.08.617237; this version posted March 15, 2025. The copyright holder for this preprint (which was not certified by peer review) is the author/funder, who has granted bioRxiv a license to display the preprint in perpetuity. It is made available under aCC-BY 4.0 International license.

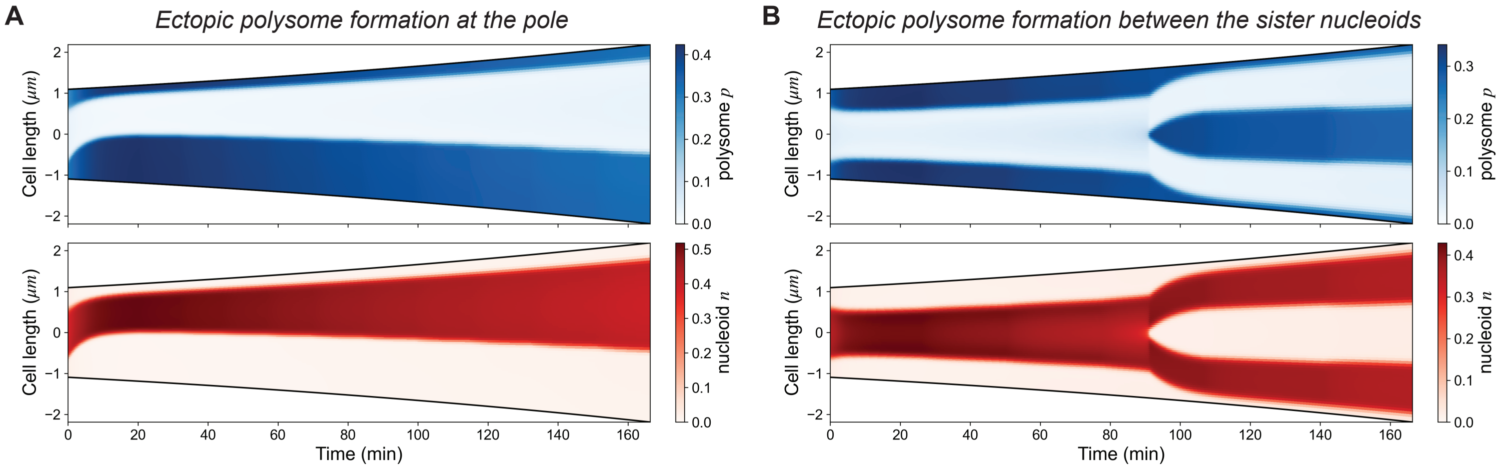

Figure 7 - figure supplement 2

bioRxiv preprint doi: https://doi.org/10.1101/2024.10.08.617237; this version posted March 15, 2025. The copyright holder for this preprint (which was not certified by peer review) is the author/funder, who has granted bioRxiv a license to display the preprint in perpetuity. It is made available under aCC-BY 4.0 International license.

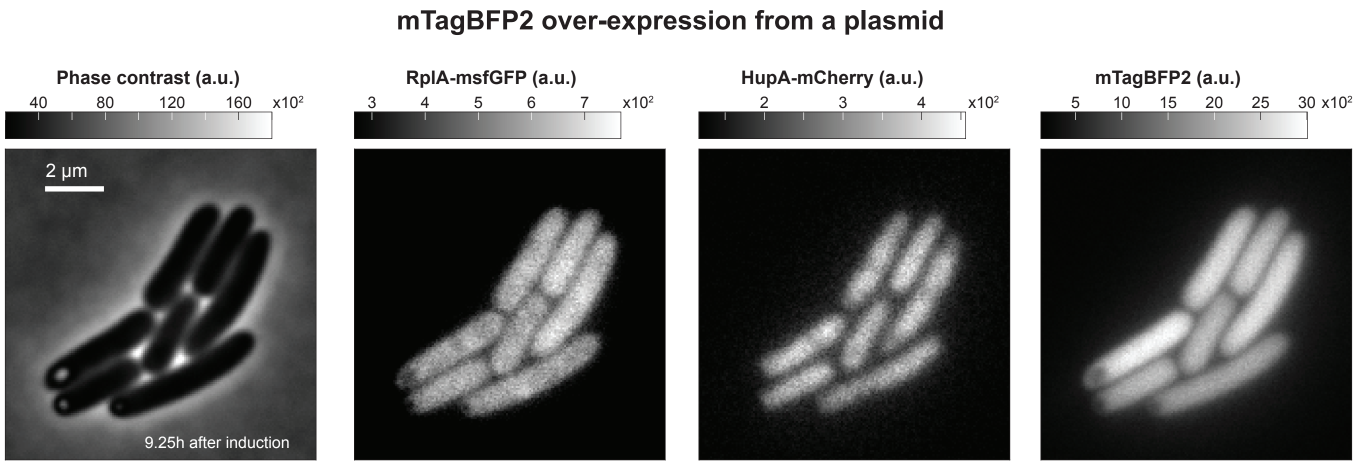

Figure 7 - figure supplement 3

bioRxiv preprint doi: https://doi.org/10.1101/2024.10.08.617237; this version posted March 15, 2025. The copyright holder for this preprint (which was not certified by peer review) is the author/funder, who has granted bioRxiv a license to display the preprint in perpetuity. It is made available under aCC-BY 4.0 International license.

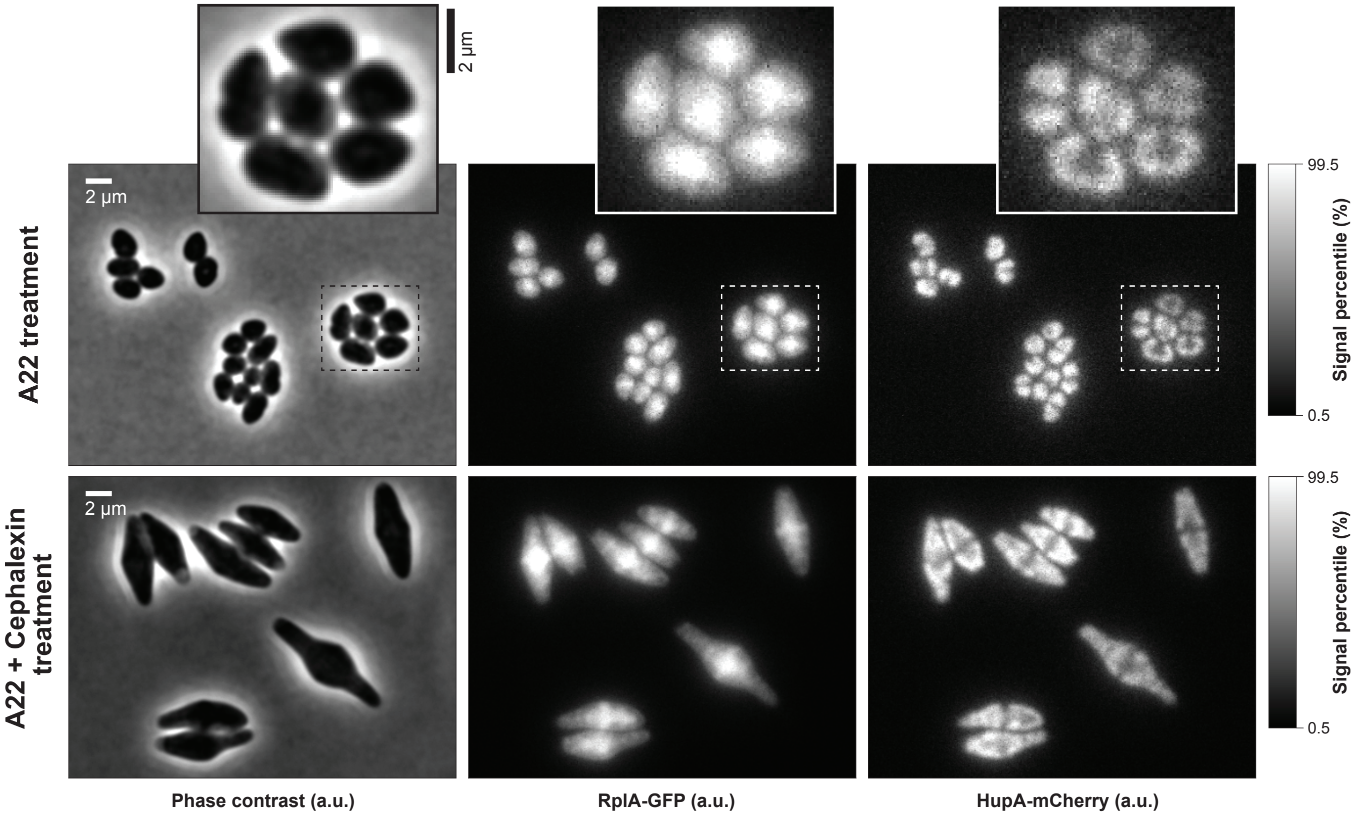

Figure 8 - figure supplement 1
